# Research on Low-Cost Non-Contact Vision-Based Wheel Arch Detection for End-of-Line Stage

**DOI:** 10.3390/s26010234

**Published:** 2025-12-30

**Authors:** Zhigang Ding, Mingsheng Lin, Yi Ding, Yun Li, Qincheng Zhang

**Affiliations:** School of Mechanical and Automotive Engineering, Fujian University of Technology, Fuzhou 350118, China; dzgsydy@fjut.edu.cn (Z.D.);

**Keywords:** machine vision, wheel arch detection, non-contact measurement, low cost, vehicle end-of-line

## Abstract

To address the collaborative requirements of high precision, high efficiency, low cost, and non-contact measurement for wheel arch detection in the calibration of Advanced Driver Assistance Systems (ADAS) during vehicle production, this study proposes a monocular machine vision-based detection methodology. The hardware system incorporates an industrial camera, priced at approximately 1000 CNY, and a custom light source. The YOLOv5s model is employed for rapid localization of the wheel hub, while the MSER algorithm, in conjunction with Canny edge detection, is utilized for robust feature extraction of the wheel arch. A geometric computation model, referenced to the wheel hub, is subsequently established to quantify the wheel arch height. Experimental results indicate that, for seven vehicle models, the method achieves an average absolute error (MAE) of ≤0.25 mm, with a maximum error of ≤0.545 mm and a single measurement time of ≤3.2 s, making it suitable for a 60 JPH production line. Additionally, under lighting conditions ranging from 500 to 1500 lux and dust concentrations of ≤10 mg/m^3^, the MAE fluctuation remains within ≤0.08 mm, ensuring consistent measurement accuracy. This methodology offers a cost-effective, reliable, and fully automated solution for wheel arch detection in ADAS calibration, demonstrating strong adaptability to production lines and considerable potential for industrial applications.

## 1. Introduction

### 1.1. Background and Motivation

The vehicle End-of-Line Stage represents a critical final step in the automotive manufacturing process, during which essential tasks such as Advanced Driver Assistance Systems (ADAS) sensor calibration and vehicle dimensional inspection must be completed. Among these, the wheel arch—defined as the vertical distance from the bottom edge of the wheel arch to the ground—serves as a fundamental vehicle reference parameter. Its measurement accuracy directly affects the reconstruction of the ADAS coordinate system. According to Chinese national standards GB/T 39263-2020 [1] and GB/T 40429-2021 [2], the measurement error of wheel arch during ADAS calibration must be controlled within ±1.0 mm. Deviations beyond this tolerance can induce spatial positioning errors in millimeter-wave radar and surround-view cameras, thereby degrading the environmental perception performance of ADAS.

The vehicle End-of-Line Stage is characterized by high production throughput, cost sensitivity, and strict non-contact inspection requirements. Specifically, production cycle times often exceed 60 JPH (60 Jobs Per Hour), per-station equipment budgets must be maintained below 100,000 CNY, and vehicle paint rework rates must remain under 0.1%. These constraints generate three critical demands for wheel arch measurement technologies: non-contact measurement to prevent paint surface damage, low-cost implementation to avoid reliance on expensive inspection devices, and automation with high efficiency, requiring single-cycle measurements to be completed in less than 10 s without manual intervention [3]. Currently, wheel arch measurement has become a key bottleneck limiting both the end-of-line throughput and ADAS calibration accuracy, highlighting the need for dedicated detection solutions suited to this industrial scenario.

### 1.2. Existing Approaches and Limitations

Extensive research has been conducted in non-contact measurement, visual detection algorithms, and intelligent manufacturing system integration, providing a solid technical foundation for this study. Accurate reconstruction of the vehicle coordinate system is fundamental to ADAS calibration, relying on precise measurement of critical reference points. Ding et al. [4] developed a machine vision-based online vehicle coordinate reconstruction method, emphasizing the pivotal role of reference point accuracy in ADAS performance. In precision dimensional measurement, Du et al. [5] utilized binocular vision to achieve high-accuracy measurement of large-diameter components, demonstrating the potential of visual geometric methods. Multi-sensor fusion has also been explored to enhance precision and robustness in vehicle contour measurement. For instance, Steinemann et al. [6] employed 3D-LIDAR to determine vehicle outlines, while Bai et al. [7] combined vision and laser point clouds to measure complete vehicle dimensions.

To meet diverse industrial measurement challenges, non-contact measurement techniques have been continually extended. Wu et al. [8] reviewed computer vision and radar-based non-contact measurement methods, emphasizing challenges related to environmental adaptability. Qian et al. [9] applied binocular vision to measure material stack heights in agricultural vehicles, and Li et al. [10] investigated optical CMM techniques for efficient measurement of complex components. Furthermore, non-contact measurement has been extended to dynamic physical quantities: Wen et al. [11] measured wheel-rail contact forces of heavy-duty trains via collaborative calibration algorithms, while Xu [12] integrated machine vision with displacement influence lines to assess vehicle load, demonstrating the versatility of these techniques from static geometrical to dynamic mechanical measurements.

Algorithmic advancements have been crucial for enhancing automation, intelligence, and robustness. In defect and target recognition, Zhu et al. [13] employed an improved Faster R-CNN for online wheel hub defect detection, while Liu et al. [14] used an enhanced YOLOv5 model for lane type recognition, highlighting deep learning’s effectiveness in complex visual tasks. For feature extraction and contour localization, both traditional algorithms and deep learning models have been investigated. Liu et al. [15] combined classical contour algorithms with machine vision for external thread measurement, Jia et al. [16] studied edge extraction under histogram equalization, and Huang et al. [17] compared traditional HSV + Canny and deep learning HED network models. U-Net-based contour extraction for workpieces has been explored by Guo et al. [18], and Lin [19] applied deep learning for wheel hub weld detection and localization. Additionally, specialized measurement methods have been developed: Liu et al. [20] proposed ring-section inspection using line-structured light, and Pondech et al. [21] combined adaptive gray mapping with particle swarm optimization for nut diameter measurement.

Integrating advanced inspection technologies into production lines is key for Industry 4.0. Yang [22] designed a machine vision system for wheel hub inspection, Chen et al. [23] developed an online measurement system for hub aperture, and Chen et al. [24] implemented precision measurement systems for component dimensions. At the system architecture level, Yu et al. [25] developed an intelligent vehicle dimensional measurement system based on structured light and machine vision, while Maślanka et al. [26] explored machine vision integration with PLC control for scalable quality inspection. Yuan et al. [27] further studied vision-based vehicle detection algorithms for ADAS, demonstrating practical applications of vision technology at the complete vehicle level.

Despite these advances, existing technologies face systemic limitations when applied to wheel arch measurement at the vehicle End-of-Line Stage, which demands ±1 mm accuracy, cycle time < 10 s, low-cost implementation, and robustness against lighting, dust, and other environmental interferences. Manual contact measurement (tape measures with reference targets) is inexpensive (~several hundred CNY) but exhibits errors >±2 mm, requires >30 s per wheel arch, and risks paint surface damage [28]. Coordinate measuring machines (CMMs) can achieve ±0.05 mm accuracy, but costs exceed 500,000 CNY, require controlled environments, and measurement time >10 min, unsuitable for high-throughput end-of-line inspection [29]. Laser profilometers and binocular vision systems avoid paint damage, yet laser devices cost > 150,000 CNY per unit, and binocular systems require expert calibration with poor model adaptability. Under workshop lighting fluctuations (500–1500 lx) and dust interference, measurement errors can rise from ±1.0 mm in laboratory conditions to ±2.0 mm, compromising process stability [30,31]. Hence, current measurement solutions fail to achieve the simultaneous combination of non-contact, low-cost, high-efficiency, and high robustness, leaving a clear gap for end-of-line wheel arch inspection.

### 1.3. Proposed Method and Contributions

To address these challenges, this study proposes a low-cost, non-contact, monocular machine vision method for wheel arch measurement. The specific contributions are as follows: a cost-effective hardware system using a DMK33GX183 industrial camera (≈1000 CNY) and an OPT-RL2416 LED light source, keeping total hardware cost under 50,000 CNY; a robust detection algorithm employing YOLOv5s for automatic wheel hub localization (mAP@0.5 = 0.995, single-frame processing time < 28 ms) combined with maximum stable extremum region segmentation and Canny edge detection to extract wheel arch features and establish a pixel-to-real-world conversion model; and scene-based experiments conducted in a real vehicle end-of-line environment with simulated lighting and dust variations across seven vehicle models (2 sedans, 5 SUVs), with 100 repeated measurements per model. Results indicate measurement accuracy within ±1.0 mm and single-cycle time < 10 s, achieving fully automated inspection without human intervention. This method provides a low-cost, high-precision, fully automated solution for wheel arch inspection in end-of-line operations, supporting intelligent ADAS calibration processes and advancing smart automotive manufacturing.

## 2. Key Technical Theory Principles

### 2.1. Monocular Camera Imaging Model

**Figure 1:**
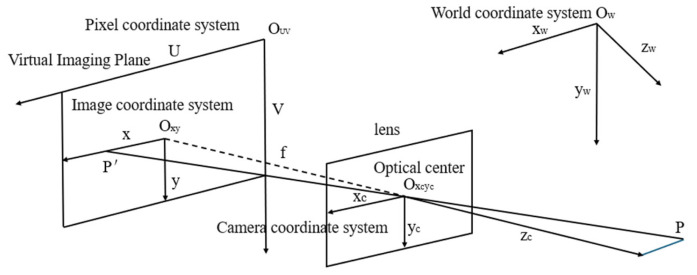
Pinhole Imaging Model Diagram.

The monocular imaging model [32] is built on the pinhole camera model, whose core objective is to establish an accurate mapping between the 3D world and 2D image. This is realized by defining four coordinate systems as follows:

1. World coordinate system (O_w_): Its origin is set at the reference point on the workshop floor, directly beneath the vehicle’s front-left wheel. The *Z*-axis is perpendicular to the ground (pointing upward) and aligned with the wheel arch measurement direction; the *X*-axis is parallel to the vehicle’s forward direction; the *Y*-axis is horizontal and perpendicular to the *X*-axis, and it describes the actual spatial position of the top vertex beneath the wheel arch.
2. Camera coordinate system (O_c_): Its origin is located at the optical center of the camera. The *Z*-axis points toward the measured vehicle along the optical axis, while the X- and Y-axes are parallel to those of the world coordinate system, acting as a transition between the two coordinate systems.
3. Image coordinate system (O_uv_): Its origin is the geometric center of the imaging plane, with the X- and Y-axes parallel to the corresponding axes of the camera coordinate system (unit: mm), reflecting the target’s physical position on the imaging plane. Note that this model differs from standard image processing conventions here: in this system, the *X*-axis points left, the *Y*-axis points downward, whereas the standard convention usually defines the *X*-axis as pointing right (with the *Y*-axis still pointing downward).
4. Pixel coordinate system (U-V): Its origin is the top-right corner of the image, with the U-axis pointing horizontally left and the V-axis pointing vertically downward (unit: pixel). This system serves as the direct coordinate reference for computer image processing, as illustrated in Figure 1.

According to the principle of similar triangles, the mapping relationship between any spatial point P(X_w_,Y_w_,Z_w_) in the world coordinate system and its corresponding projection point p(u,v) in the pixel coordinate system can be expressed as:

(1) $$\left[\begin{array}{c} u\\ v\\ 1 \end{array}\right]=\frac{1}{Z_{w}}K\left\{R|t\right\}\left[\begin{array}{c} X_{w}\\ Y_{w}\\ \begin{array}{c} Z_{w}\\ 1 \end{array} \end{array}\right]$$

In the formula, K is the camera intrinsic matrix containing the focal lengths (fx, fy) and the principal point coordinates (u0, v0); R is the 3 × 3 rotation matrix; t is the 3 × 1 translation vector; and (1/Z_w_) is the scaling factor used to eliminate the scale ambiguity in the 3D-to-2D projection.

### 2.2. Low-Cost Camera Calibration

Camera calibration, as a core prerequisite for obtaining the intrinsic and extrinsic parameters of a monocular measurement system, directly determines the accuracy and robustness of subsequent wheel arch measurements [33]. Traditional checkerboard calibration methods rely on high-precision printed checkerboards, requiring a manufacturing accuracy of ≤0.02 mm. Moreover, the surface of the checkerboard is susceptible to reflections from workshop lighting, which can cause corner extraction deviations, making it difficult to adapt to complex detection environments. To address this, this study adopts an aluminum circular calibration board to build a low-cost, highly robust calibration solution. The specific design is as follows: The calibration board follows the Chinese standard GB400-20-7×7 specification, with a 4 mm center-to-center distance between adjacent feature points. The manufacturing accuracy is controlled within ±0.01 mm, and the cost of a single calibration board is only one-fifth that of a high-precision checkerboard calibration board with the same accuracy. The metal material offers resistance to dust contamination and wear, reducing interference from dust adhesion on feature extraction. Additionally, compared to checkerboard corners, circular feature points effectively avoid recognition ambiguity caused by edge blurring or uneven lighting. Under workshop lighting conditions fluctuating between 500 and 1500 lux, the success rate of circular feature point extraction is ≥98%, significantly outperforming the 85% success rate of checkerboard corner extraction, with a 15% improvement in feature extraction accuracy.

**Figure 2:**
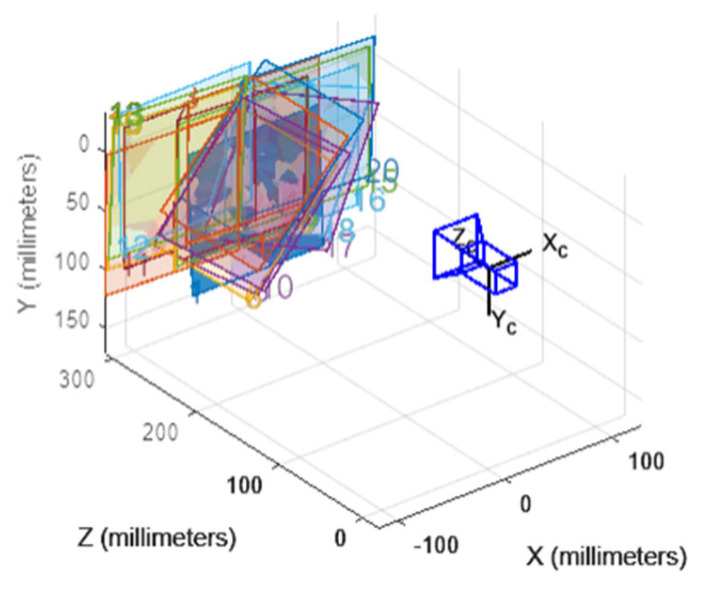
Camera Calibration Diagram.

**Figure 3:**
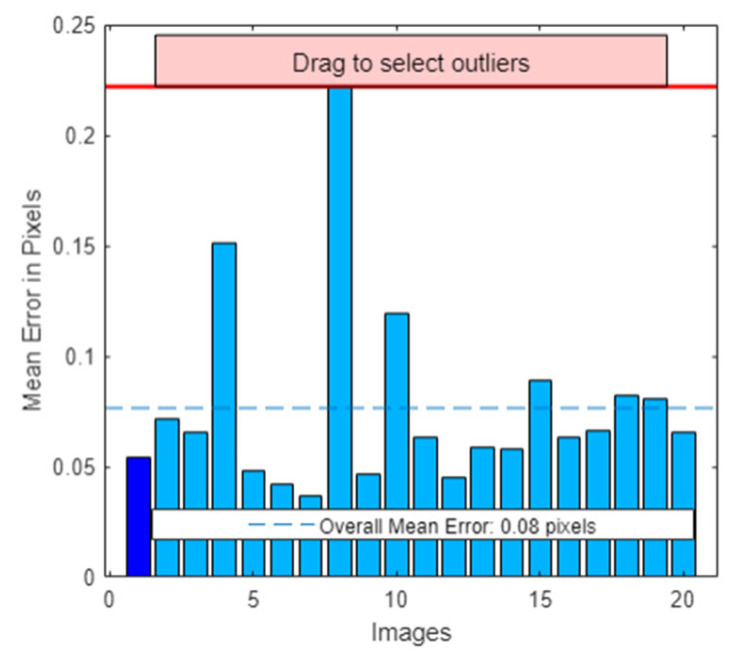
Calibration Error Histogram.

The specific steps are as follows: (1) Fix the camera on the preset mounting bracket at the inspection station to ensure that the camera’s posture and height are perfectly aligned with those in the actual detection setup, thereby preventing calibration parameter errors due to installation deviations; (2) Adjust the spatial angle and position of the aluminum circular calibration plate to fully cover the camera’s field of view. A total of 20 calibration images are taken, with the angular interval between the calibration plate positions in adjacent images controlled within 5° to 10° to avoid parameter redundancy; (3) The MATLAB 2022b Camera Calibration Toolbox is used to calculate the calibration parameters, and a sub-pixel-level circle center extraction algorithm based on the grayscale center of gravity method is introduced during feature point extraction to further optimize the accuracy of feature point positioning. The calibration results indicate that the average reprojection error of the system is ≤0.3 pixels, which ensures that the system error in subsequent wheel arch measurements is controlled within ±0.1 mm, as shown in Figure 2 and Figure 3, fully meeting the parameter accuracy requirements for wheel arch detection.

The camera intrinsic parameter matrix includes the equivalent focal length (FX, FY) and the principal point coordinates (U0, V0), which provides core parameter support for the conversion of pixel coordinate system to image coordinate system, and is directly used for the subsequent conversion of pixel information and actual physical size. The external parameters quantitatively reflect the relative attitude and translation relationship between the camera coordinate system and the world coordinate system, which is essential for the accurate positioning of the ground reference in the height measurement of the wheel arch. Finally, the camera intrinsic parameter matrix A and the distortion coefficients k are also solved.

### 2.3. Camera Distortion Correction

Low-cost industrial camera lenses are subject to optical design simplifications and manufacturing cost constraints, often exhibiting two types of optical distortions: radial distortion and tangential distortion. These distortions can lead to pixel-level displacements of the wheel arch edges in images, particularly causing positioning errors of key feature points—such as the lower edge vertices of the wheel arch—that can reach 2 to 3 pixels. This, in turn, reduces feature extraction accuracy and ultimately impacts the precision of wheel arch calculations. Therefore, it is essential to compensate for these distortions by constructing a simplified distortion correction model. The primary objective is to ensure that the correction accuracy meets the ±0.5 mm measurement requirement, while also reducing the algorithmic computational complexity to accommodate real-time detection needs.

**Figure 4:**
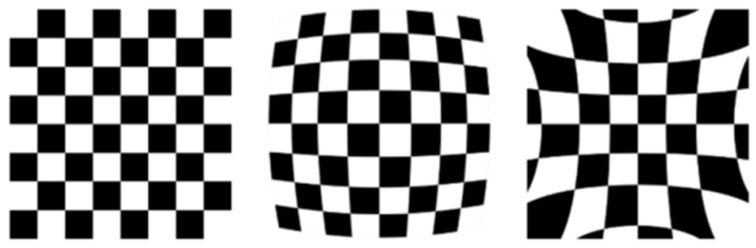
Radial Distortion Diagram.

Radial distortion, in particular, arises from the uneven distribution of the refractive index in the lens optics and assembly errors, and it typically manifests as geometric distortion in areas farther from the optical center of the image, as illustrated in Figure 4. To balance correction accuracy and computational efficiency, this study employs a polynomial radial distortion model with three terms, including coefficients up to k_3_ (third-order radial distortion), to compensate for radial distortion. Compared with higher-order polynomial models, the proposed model reduces the computational load during distortion correction by approximately 40%, while ensuring that the residual radial distortion error remains within 0.1 pixels. This effectively prevents production delays that would otherwise exceed the vehicle-level requirement of 60 JPH due to prolonged computation times. The correction principle involves establishing a mapping relationship between the distorted pixel coordinates and the ideal pixel coordinates, which can be described by Equations (2) and (3):

(2) $$x^{\prime }=x\left(1+k_{1}r^{2}+k_{2}r^{4}+k_{3}r^{6}\right)$$

(3) $$y^{\prime }=y\left(1+k_{1}r^{2}+k_{2}r^{4}+k_{3}r^{6}\right)$$

Tangential distortion arises from misalignment or tilt between the lens elements, leading to a distortion of objects in the image, causing them to appear skewed or slanted. Straight lines may appear to be tilted at certain angles in the image. The tangential distortion model is:

(4) $$x^{\prime }=x+\left[2p_{1}xy+p_{2}\left(r^{2}+2x^{2}\right)\right]$$

(5) $$y^{\prime }=y+\left[2p_{2}xy+p_{1}\left(r^{2}+2y^{2}\right)\right]$$

Integrating Equations (4) and (5) gives the overall distortion model formula for radial and tangential distortions.

(6) $$x^{\prime }=x+x\left(k_{1}r^{2}+k_{2}r^{4}+k_{3}r^{6}\right)+\left[2p_{1}xy+p_{2}\left(r^{2}+2x^{2}\right)\right]$$

(7) $$y^{\prime }=y+y\left(k_{1}r^{2}+k_{2}r^{4}+k_{3}r^{6}\right)+\left[2p_{2}xy+p_{1}\left(r^{2}+2y^{2}\right)\right]$$

where $r^{2}=x^{2}+y^{2}$, (x, y) are the uncorrected pixel coordinates, and (x′, y′) are the corrected pixel coordinates. k_1_ to k_3_ are the coefficients of radial distortion, and p_1_ and p_2_ are coefficients of tangential distortion.

**Figure 5:**
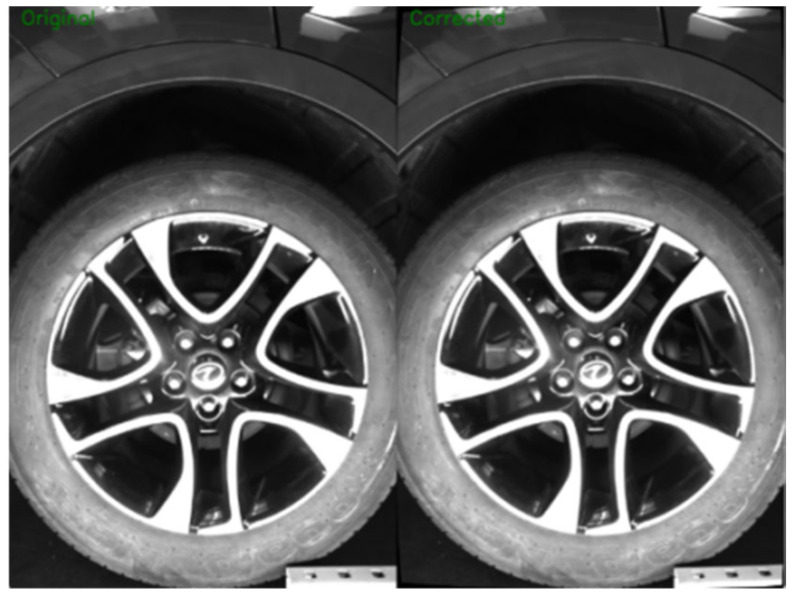
Comparison of Wheel Arch Corrections.

To further enhance real-time detection efficiency, a precomputed mapping table method is introduced to optimize the calculations [34]. This method allows direct access to the corrected position of each pixel through table lookup during the real-time detection phase, eliminating the need for repetitive polynomial solving or other redundant calculations, thereby effectively reducing computational complexity. The correction results are shown in Figure 5.

### 2.4. Wheel Arch Calculation Model

Given the diversity of vehicle body parameters and the interference characteristics of the workshop environment in the vehicle end-of-line scenario, this study develops a wheel arch calculation model based on the center of the wheel hub as the intermediate reference. This reference point, inherent to the vehicle’s structural design, maintains a stable relative position with both the wheel arch and the ground within the same model. Consequently, there is no need for additional ground calibration marks, simplifying the detection process, avoiding environmental interference issues with the ground reference, and significantly enhancing the model’s anti-interference capability.

Traditional wheel arch measurement models typically rely on direct distance measurements from a ground reference point. These methods require the installation of high-precision ground calibration marks at the inspection station, often using metal markers for location. However, these markers are easily covered by dust or worn due to vehicle movement in the workshop, leading to increased datum positioning errors. Additionally, for vehicles with varying wheelbases and wheel diameters, the ground reference position must be repeatedly adjusted, which reduces adaptability and complicates batch testing.

**Figure 6:**
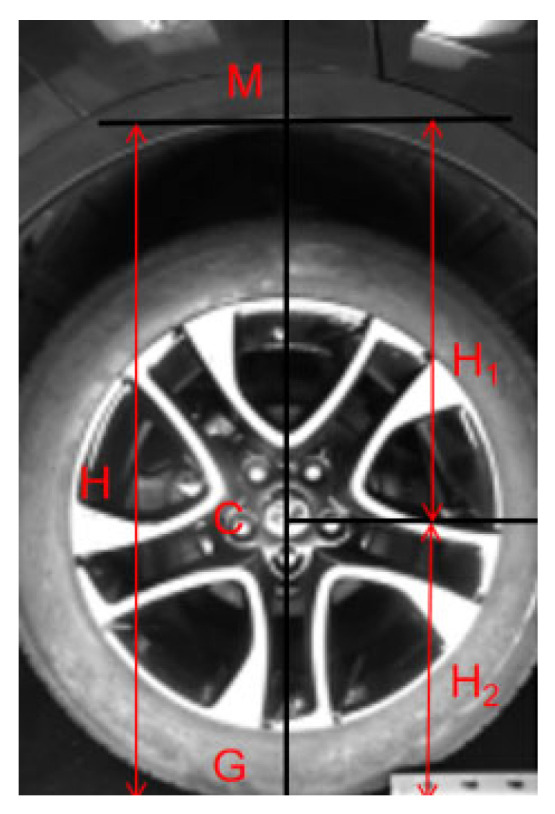
Wheel arch Calculation Model. Schematic diagram.

In contrast, this study proposes a more adaptable wheel arch calculation model, as shown in Figure 6. The model divides the measurement process into three key elements: wheel arch, wheel hubs, and the ground. The lower edge vertex of the wheel arch, denoted as point M, serves as the endpoint for height measurement. The hub center, referred to as point C, is the central reference, while the ground, represented by point G, marks the starting point of the height measurement at the contact point between the hub and the ground. The formula is derived by establishing a geometric relationship between these three points.

(8) $$H=H_{1}+H_{2}$$

In the formula, *H* represents the actual height from the wheel arch to the ground, *H*_1_ is the height from the wheel hub center to the lower edge of the wheel arch, and *H*_2_ is the distance from the wheel hub center to the ground.

Based on the monocular pinhole imaging model constructed in Section 2.1, a geometric ranging model for calculating the wheel arch is further derived: First, the coordinates of points M and C in the pixel coordinate system, (uM, vM) and (uC, vC), are obtained through image feature extraction; next, based on the intrinsic camera parameters (fx, fy) and (u_0_, v_0_) and extrinsic parameters (R, t) calibrated in Section 1.2, the pixel coordinates are converted to three-dimensional coordinates in the world coordinate system (X_M_, Y_M_, Z_M_) and (X_C_, Y_C_, Z_C_); finally, utilizing the characteristic the fact that the *Z*-axis in the world coordinate system aligns with the wheel arch direction, combined with the known vertical distance H_CG_ from the wheel hub center to the ground, during vehicle end-of-line production and when the vehicle is in a static, unloaded state with standard tire pressure, the measurement from the wheel hub center to the ground is a fixed vehicle parameter for the same model.

To correct the inherent scale errors and unmodeled system biases in the monocular vision measurement model, this study introduces a comprehensive scaling correction factor k1 and an offset correction factor b. These corrections aim to compensate for systematic errors in the pixel-to-world coordinate transformation, caused by factors such as the simplified camera model, installation misalignment, and variations in working distance. Calibration and Derivation: The calibration of these factors is performed through high-precision experiments. A PLC-controlled linear guide drives the calibrated reference fixture, which moves with 1.0 mm precision within the system’s working distance range. Images are captured at each position, and the corresponding data pairs of actual displacement and pixel displacement are collected, with more than 100 valid samples accumulated. The dataset is then subjected to linear fitting using the least squares method, yielding the optimal scaling correction factor k_1_ and offset correction factor b. This process effectively absorbs multiple system errors, including residual distortion and installation imperfections, into the two parameters.

The final corrected wheel arch calculation model is given by Equation (9).

(9) $$H=k_{1}Z_{m}+b$$

**Figure 7:**
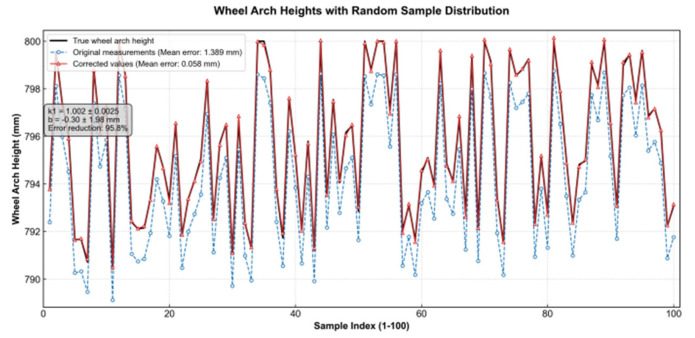
Positioning Test Calibration Result.

In this equation, Z_m_ represents the raw measurement value based on the initial camera parameters. As shown in Figure 7, the empirical correction model significantly reduces the system’s theoretical calibration error from ≤1.389 mm to ≤0.058 mm through calibration experiments. During subsequent batch validation involving seven vehicle models—two sedans and five SUVs—the corrected wheel arch measurement error is consistently controlled within ±0.3 mm. This demonstrates that the proposed compensation mechanism using k_1_ and b effectively enhances the overall accuracy and robustness of the monocular vision system in real-world workshop environments.

## 3. Design and Implementation System

### 3.1. Hardware System Design

**Figure 8:**
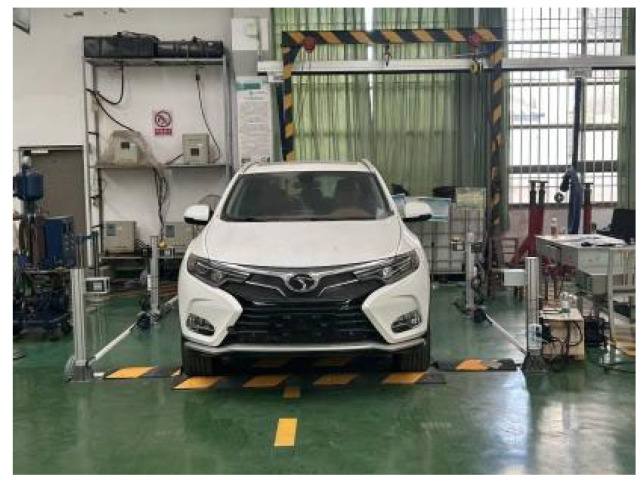
Overall Structure Diagram of Laboratory Hardware.

The hardware selection adheres to the principle of “adequate is optimal and cost is controllable,” prioritizing cost-effective components while ensuring that performance meets the accuracy and efficiency requirements for wheel arch inspection. The specific configuration is illustrated in Figure 8:

#### 3.1.1. Core Perception Module

Industrial Camera: The Imaging Source DMK33GX183 CMOS 3-megapixel camera (The Imaging Source Asia Co., Ltd., Shanghai, China) is used, offering a resolution of 2048 × 1536 pixels, a pixel size of 3.45 μm × 3.45 μm, and a maximum frame rate of 60 fps. This camera costs only one-third of a high-end industrial camera with the same resolution, such as the Basler acA2500 (Basler AG, Ahrensburg, Germany; assembled in Suzhou, China), while fully meeting the requirements for capturing wheel arch details. At a working distance of 0.6–1.2 m, the pixel width of the wheel arch edges is ≥3 pixels, ensuring high accuracy in feature extraction. The 60 fps frame rate allows for fast image capture, preventing motion blur caused by slight vehicle movements.

Lighting System: The system employs the Opt-RL2416 blue bar LED light (Opto Engineering China Co., Ltd., Shenzhen, China) source from Opt, paired with a diffuser and light shield. Blue light has a reflectivity of approximately 30% on metallic car paint, lower than the 60% reflectivity of white light, effectively reducing reflective interference. The 20 W adjustable power range (300–2000 lux) accommodates workshop lighting fluctuations between 500 and 1500 lux. The diffuser ensures lighting uniformity of ≥90%, maintaining consistent gray distribution across the wheel arch area.

#### 3.1.2. Fixing and Positioning Module

**Figure 9:**
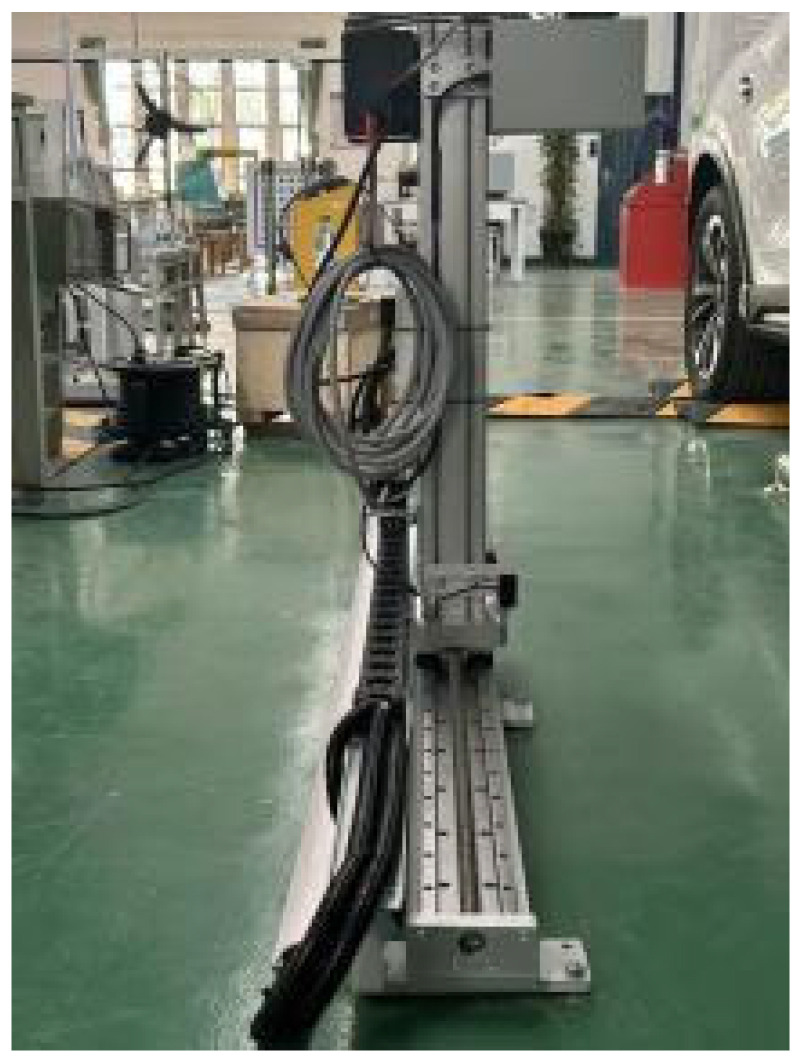
Camera Mount.

The camera bracket is constructed from 4040 aluminum profiles in an ‘L’ shape and is controlled by a PLC for rail adjustment. The adjustable range spans 800–1500 mm, fully covering the detection needs for both sedans and SUVs. The horizontal travel distance of 1500 mm enables precise alignment of the camera’s field of view with the center of the wheel arch during installation and calibration. To address workshop vibration interference, the offline adaptation design incorporates the following features: (1) The bracket’s base is rigidly connected to a 10 mm thick steel plate using M12 expansion bolts, securely fixed to the workshop floor, effectively reducing vibration transmission from conveyor operations (20–50 Hz) to below 10%; (2) A 5 mm thick nitrile rubber vibration-damping pad is added at the camera mounting point, achieving a vibration attenuation rate of ≥90%. Laser interferometer tests show that the repetitive positioning error of the camera is ≤0.1 mm, effectively preventing calibration drift due to vibration and ensuring long-term measurement accuracy. The detailed structure is illustrated in Figure 9:

Vehicle Centering Assistance: The existing roller side guide wheel device at the offline station is reused, with the guide wheels on both sides of the roller track adjusted to accommodate the wheel distance of different vehicle models. This works in conjunction with synchronized roller speed control to achieve preliminary vehicle positioning. To further enhance accuracy, an infrared beam sensor is installed at the end of the guide wheel to detect the edge position of the wheel in real time. The guide wheel is then fine-tuned by the PLC to within ±1 mm, ensuring that the horizontal deviation between the center of the wheel hub and the optical axis of the camera is ≤5 mm. This design not only ensures that the wheel arch area remains within 80% of the camera’s field of view, reducing the proportion of invalid detections from 8% to less than 0.5%, but also establishes a coordinate linkage between the centering reference and the ADAS calibration station during the vehicle off-line process. This allows for data connectivity without the need for additional calibration, ultimately improving the overall detection efficiency.

**Figure 10:**
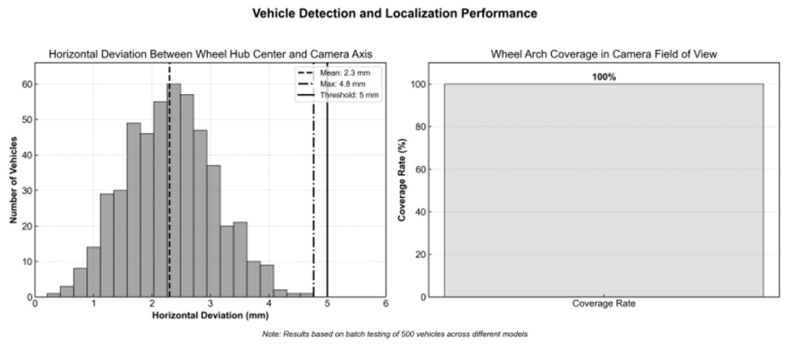
Positioning Detection Results.

After batch testing, which involved continuous detection of 500 vehicles, the average horizontal deviation between the center of the wheel hub and the optical axis of the camera was found to be 2.3 mm, with a maximum deviation of 4.8 mm, all within the 5 mm threshold. The coverage rate of the wheel arch area within the camera’s field of view was 100%, with no instances of the wheel arch exceeding the field of view due to vehicle offset. This ensures the continuity of detection, as demonstrated in Figure 10.

#### 3.1.3. Computation and Control Module

The calculation and control module serves as the data processing and coordination hub for the wheel arch detection system. An Advantech IPC-610L (Advantech Co., Ltd., Suzhou, China) open industrial computer was selected, configured with an Intel Core i5-10400 processor, 16 GB DDR4 memory, and a 512 GB SSD. This configuration meets the system’s critical requirements for “real-time performance, stability, and scalability.” In terms of computing power, the USB 3.0 interface supports single-frame image transmission at a resolution of 3 million pixels, with a transmission time ≤20 ms, ensuring no data loss at the camera’s 20 fps frame rate. The CPU can run core OpenCV algorithms in a single thread with a processing time ≤800 ms, well below the ≤5 s threshold for single detection cycles. The integrated UHD Graphics 630 GPU also accelerates YOLOv5s inference, enhancing wheel arch localization efficiency.

In terms of industrial stability, the industrial-grade motherboard can withstand workshop environments with dust concentrations of ≤10 mg/m^3^ and voltage fluctuations of 220 ± 10 V, supporting continuous operation for up to 8 h and adapting to a production pace of 60 jobs per hour (JPH). Regarding system scalability, the reserved RS485 interface allows direct communication with the production line MES system, enabling real-time uploading of detection results and closed-loop quality data management, thus supporting full-process automation from image acquisition to height output.

### 3.2. Wheel Arch Measurement Software System

**Figure 11:**
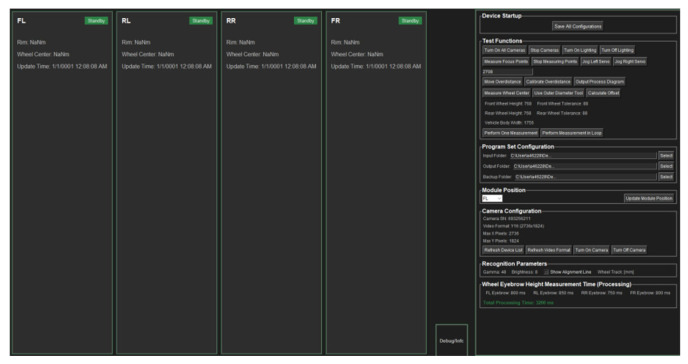
Wheel arch Detection Software Interface.

The vehicle wheel arch testing system monitoring software developed in this study adopts a modular dark-themed user interface design (Figure 11), integrating functional modules for real-time four-wheel arch status monitoring, equipment control, parameter configuration, and performance analysis. The left panel of the interface displays the rim dimensions, wheel center positions, wheel arch, and measurement status of the four wheels, namely front-left (FL), rear-left (RL), rear-right (RR), and front-right (FR). The right-side control panel provides a complete operational workflow, including camera and illumination control, focus measurement, and servo adjustment.

To enhance efficiency under vehicle end-of-line conditions, a wheel arch measurement time statistics module is innovatively integrated, enabling real-time visualization of processing time at each measurement point. Through parallel task scheduling and data buffering strategies, the total processing time is stably controlled within 3200 ms, which is significantly lower than the vehicle end-of-line takt time requirement. The software is developed based on Python 3.8, leveraging open-source libraries such as OpenCV and PyTorch, (Version 1.13.1) and demonstrates high real-time performance, good maintainability, and low implementation cost. Validation on an actual production line confirms that the system achieves stable measurement accuracy and reliable operational performance.

#### 3.2.1. Wheel Hub Detection Based on YOLOv5s

To enhance the adaptability of the wheel arch detection system to mixed-model production during vehicle end-of-line operations and to enable accurate identification of wheel arch regions as well as reliable body parameter extraction across different vehicle models, a core perception module was constructed based on the framework described in Section 3.1.1. Under shooting angles ranging from 0° to 15° and illumination conditions between 500 and 1500 lux, a dataset comprising 1000 high-resolution images was collected from seven vehicle models, including both sedans and SUVs. The dataset was divided into a training set (80%, 800 images) and a testing set (20%, 200 images).

Wheel hub bounding boxes, vehicle model categories, and center coordinates were annotated using LabelImg and subsequently converted into the YOLO format. To improve generalization performance, data augmentation techniques—including horizontal flipping, rotation within −10° to +10°, and brightness perturbations of ±20%—were applied to the training set. A lightweight YOLOv5s network was selected as the detection model, and transfer learning was implemented using COCO pre-trained weights. The model was trained for 200 epochs with a batch size of 16, employing the Adam optimizer with an initial learning rate of 0.001, which was gradually reduced to 0.0001 using a cosine annealing schedule. The CIoU loss function was adopted, and early stopping was applied to mitigate overfitting.

**Figure 12:**
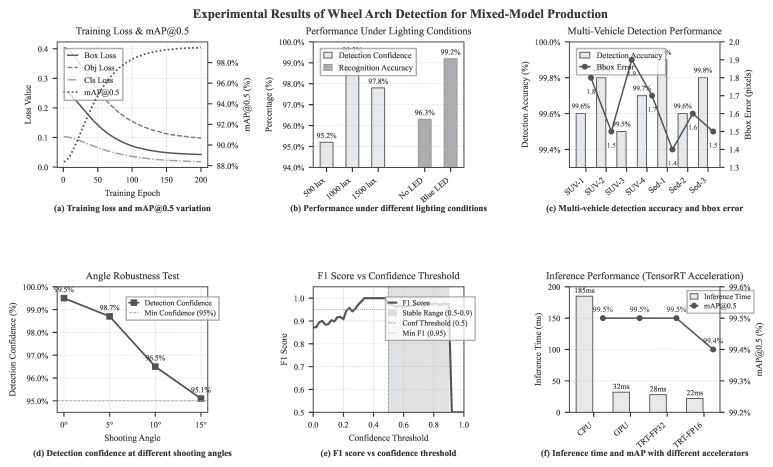
Deep Learning Training Results.

After training, the classification accuracy reached 99.8%. Evaluation on the testing set demonstrated that the YOLOv5s model [35] achieved an mAP@0.5 of 0.995, with 100% vehicle model recognition accuracy and a wheel hub bounding box localization error of no more than 2 pixels. With TensorRT acceleration, the single-frame inference time was maintained below 28 ms. Under challenging conditions involving 500 lux illumination and a 15° shooting angle deviation, the detection confidence remained above 0.95. Furthermore, after suppressing surface reflections using a blue LED light source, the overall detection accuracy remained at 99.2%. The experimental results, as illustrated in Figure 12, confirm the high reliability and strong scene adaptability of the proposed approach, providing a robust foundation for subsequent wheel arch feature extraction and height measurement.

#### 3.2.2. Image Preprocessing for Robust Wheel Arch Detection

To suppress interference caused by workshop dust, illumination fluctuations, and vehicle body reflections, and to enhance the robustness of wheel arch region feature recognition for subsequent wheel hub localization and wheel arch feature extraction, a four-stage image preprocessing pipeline—namely denoising, enhancement, cropping, and adaptation—was designed. The effects of each preprocessing stage on image quality, processing efficiency, and detection performance were quantitatively evaluated. All experiments were conducted using workshop images with a resolution of 2048 × 1536.

After applying multi-scale Gaussian filtering for noise suppression and a Retinex–CLAHE [36] fusion enhancement strategy [37], the image signal-to-noise ratio (SNR) increased from 28 dB to 42 dB, while the background gray-level contrast in the wheel arch region increased from 18 to above 30. Meanwhile, the effective data volume was reduced to 95% of the original size through region cropping and adaptive processing. As a result, the wheel arch detection accuracy improved from 89.2% to over 99.5%, and the false detection rate was reduced from 5.7% to below 0.3%. Furthermore, the proposed preprocessing scheme demonstrated compatibility with approximately 98% of workshop operating conditions, fully satisfying the robustness and reliability requirements of industrial inspection.

#### 3.2.3. Wheel Hub Center Feature Point Extraction and Error Compensation

As a critical reference point for wheel arch measurement, the localization accuracy of the wheel hub center directly determines the precision of the final inspection results. Under vehicle end-of-line conditions, wheel hub center extraction is often affected by workshop illumination fluctuations, dust contamination, and surface reflections. Current mainstream approaches for wheel hub center detection include Canny edge detection, circle fitting, Hough transform-based methods, deep learning-based detection, and template matching techniques [38,39].

**Figure 13:**
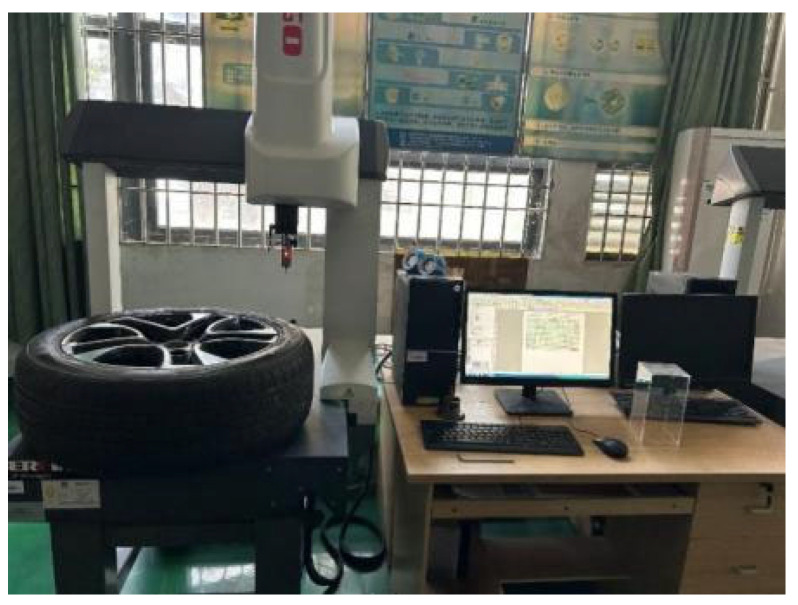
Diagram of the Hub Diameter Reference Value Measuring Device.

To evaluate the adaptability and accuracy of different methods under industrial conditions, comparative experiments were conducted using wheel diameter reference values measured by a coordinate measuring machine (CMM), as illustrated in Figure 13. The processing results obtained using threshold segmentation, the Maximum Stable Extremal Region (MSER) method, and the Canny edge detection method are summarized in Table 1. The experimental results indicate that the wheel hub center extracted using the MSER method exhibits higher positioning accuracy than those obtained by the other two approaches. Specifically, the MSER-based method achieves the smallest measurement deviation, with an error of only −0.391 mm relative to the reference value, while demonstrating superior robustness against illumination variations.

The MSER algorithm, known for its robustness under varying lighting conditions and its ability to handle noise interference, was chosen for wheel hub center feature point extraction. This algorithm has been extensively shown to perform well in environments with fluctuating illumination and dust contamination [40]. Additionally, the combination of MSER with morphological processing significantly enhances feature extraction by eliminating noise and improving the precision of the wheel hub center localization. Morphological operations, such as dilation and erosion, help clarify the boundaries of the wheel hub and improve the accuracy of center extraction [41].

The wheel hub center extracted with a −0.391 mm error will propagate through the height measurement process. However, the error propagation in this case is controlled through a combination of geometric calibration and error compensation models. Since the wheel arch measurement is based on the relative positions of the wheel hub center and the wheel arch, small errors in hub center localization have a limited effect on the final height measurement. The error is minimized by:

1. Geometric Calibration: Accurate calibration of the camera and system ensures that even small deviations in hub center position do not result in significant errors in the final measurements.
2. Error Compensation: A compensation algorithm is employed to correct small errors caused by wheel hub center localization. This ensures that the final wheel arch measurement remains within the required ±1.0 mm precision.

Therefore, the −0.391 mm error in the hub center positioning has a minimal impact on the final wheel arch measurement, maintaining the required accuracy and ensuring the reliability of the system.

**Figure 14:**
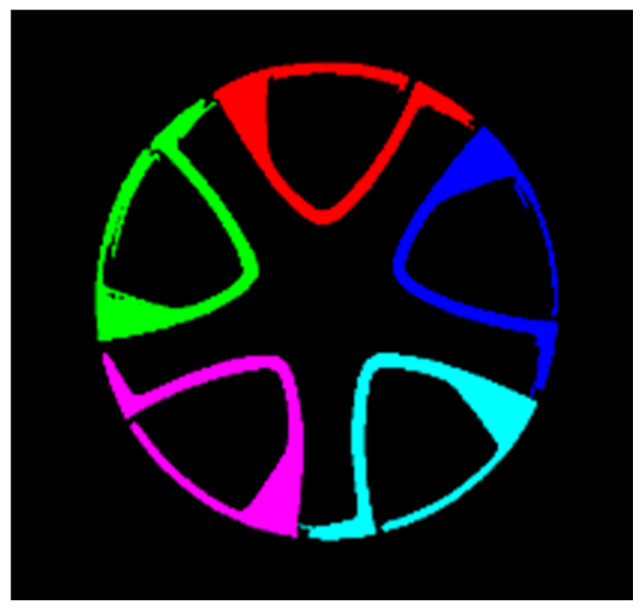
Wheel Hub Area Extraction Diagram.

**Figure 15:**
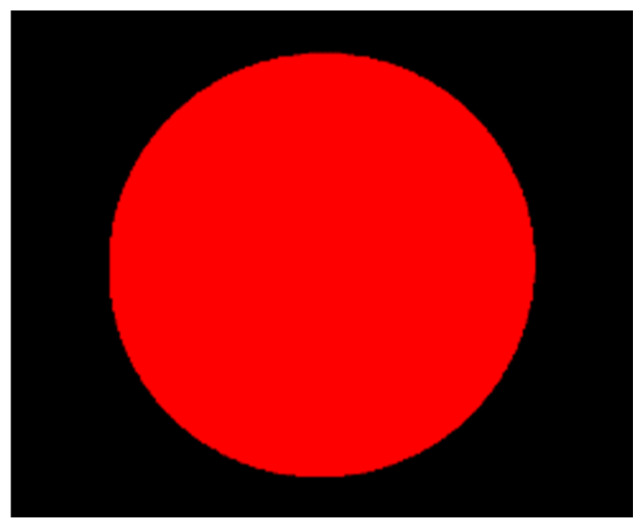
Hole Filling Processing Diagram.

**Figure 16:**
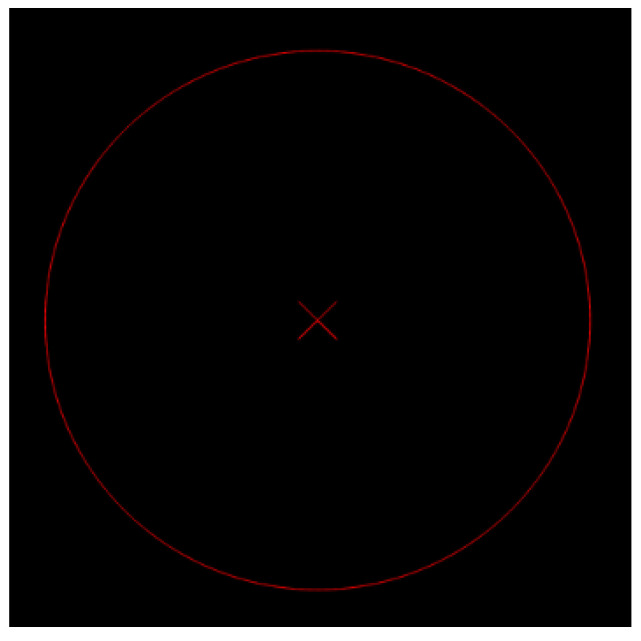
Extraction of Circle Center Result.

The specific steps for hub area detection and center positioning are as follows: First, grayscale conversion transforms the color image (after histogram equalization in Section 3.2.2) into a grayscale image, with the conversion formula shown in Equation (10). Then, the “segmentation-optimization-fitting” process is followed:

1. Segmentation: Based on the watershed concept, the MSER algorithm, which utilizes affine invariance and grayscale robustness, is used to calculate regional stability according to Equation (11) to segment and obtain the initial hub region (Figure 14).
2. Optimization: The cv2.bitwise() function is called to merge discrete hub regions. Then, cv2.floodFill() is used to fill holes within the region, eliminating interference from hub spokes, resulting in the complete hub region (Figure 15).
3. Fitting: Finally, the cv2.minEnclosingCircle() function is used to fit the minimum enclosing circle to the complete region, outputting the hub center coordinates and radius (Figure 16), with the final center positioning error ≤ 0.39 mm, meeting the baseline accuracy requirement.

(10) Gray(i,j)=max{R(i,j),G(i,j),B(i,j)}

In the formula, Gray(i,j) is the grayscale value at pixel (i,j) after conversion, and R(i,j), G(i,j), B(i,j) are the red, green, and blue brightness values at pixel (i,j) in the original image, respectively.

(11) $$q(i)\frac{\left|R\left(i+\Delta \right)-R(i)\right|}{\left|R(i)\right|}$$

In the formula, R(i) represents a certain connected region at threshold i, Δ is a small increment of the grayscale threshold, q(i) is the rate of change in region R(i) when the threshold is i, and |R(i)| denotes the area of region R(i).

#### 3.2.4. Wheel Arch Edge Contour Extraction for Height Calculation

**Figure 17:**
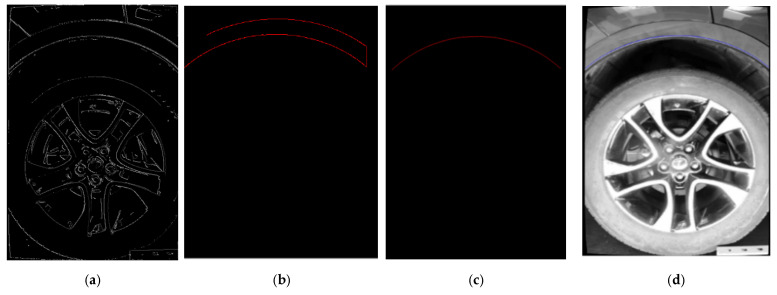
Edge Contour Extraction Process of Wheel Arch. (**a**) Contour extraction via threshold segmentation. (**b**) Wheel arch contour filtering. (**c**) Wheel arch contour extraction. (**d**) Display of wheel arch contour on the original image.

The extraction of wheel arch edge contours is critical for obtaining feature points used in height calculation. This process is implemented using the Canny edge detection algorithm combined with region filtering [42], as shown in Figure 14, Figure 15 and Figure 16:

1. Edge Detection: The grayscale image obtained in the previous section is used as input. The Canny algorithm is applied with double thresholds of 60/180 and an aperture size of 3 to extract all edges in the image, resulting in an edge distribution map (Figure 17a).
2. Region Filtering: A wheel arch-specific Region of Interest (ROI) is defined based on the wheel hub center position to filter out irrelevant edges, such as those from car doors and bumpers. This leaves only the wheel arch edges (Figure 17b).
3. Contour Segmentation: The filtered wheel arch edges are segmented into line segments. Short noise lines, shorter than 50 pixels, are discarded, while the approximate vertical main edges of the wheel arch, with an orientation angle of 80–100°, are retained. This results in the wheel arch edge contour line segments (Figure 17c).
4. Result Output: The final wheel arch edges are marked with blue contour lines (Figure 17d), providing clear feature boundaries for subsequent height calculation.

#### 3.2.5. Wheel Arch Calculation and Measurement Algorithm

**Figure 18:**
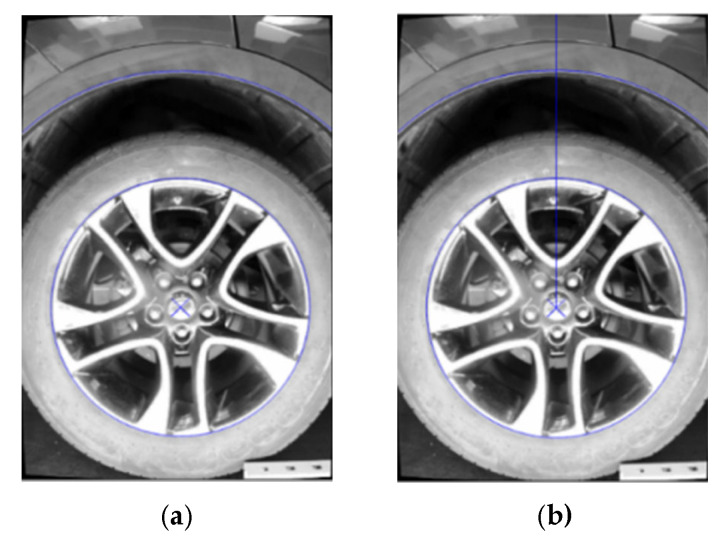
Wheel arch Edge Computing Processing Diagram. (**a**) Schematic Diagram of Feature Extraction. (**b**) Schematic Diagram of Synthetic Region.

Calculation of the distance from the wheel hub center to the wheel arch edge is achieved through the superimposition of visual features for precise measurement. First, the wheel hub center point extracted in Section 3.2.2 (shown in Figure 16, denoted as center c (Xc, Yc)) is pixel-level aligned with the wheel arch contour shown in Figure 17c, and merged to obtain Figure 18a; then, a vertical line is drawn from center C to the ground, obtaining the intersection point G(Xg, Yg) with the wheel arch edge (Figure 18b), and the vertical pixel distance between the two points is calculated as shown in Equation (12):

(12) $$\Delta y=|y_{g}-y_{c}|$$

Finally, by using the pixel-to-physical size coefficient k, which is calibrated based on the wheel hub diameter, the actual distance from the center of the wheel hub to the edge of the wheel arch is calculated by substituting into Equation (13).

(13) $$h_{1}=\Delta y\times k$$

**Figure 19:**
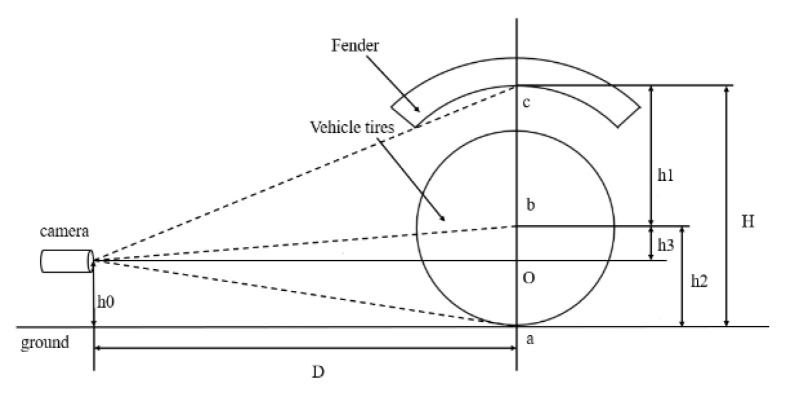
Wheel Hub Center to Ground Height Measurement Model.

Since the calculation of the distance from the wheel hub center to the ground cannot directly determine three-dimensional depth with monocular vision alone, it must be indirectly derived using known physical references. Referring to Figure 19 as a reference, without considering camera installation errors, let the optical axis center O coincide with the origin of the camera coordinate system, with an initial height h_0_ from the ground; then, the actual vertical distance d from O to the wheel hub center b can be calculated through the camera intrinsic matrix. Finally, according to the geometric relationships, as shown in Equation (14):

(14) $$h_{2}=h_{0}-d$$

The actual distance from the wheel hub center to the ground is then obtained. Finally, the actual height of the wheel arch is optimized based on error compensation and the correction coefficient Equation (9), completing the wheel arch detection.

## 4. Experimental Results and Analysis

To verify the accuracy, vehicle adaptability, and environmental robustness of the wheel arch detection method in a full-vehicle offline scenario, experiments were conducted using a dual-scenario approach: ‘laboratory-simulated interference + on-site production line adaptation.’ The reliability of the system was evaluated by constructing a standardized platform, collecting multi-vehicle data, and quantifying error indicators.

### 4.1. On-Site Platform Construction

**Figure 20:**
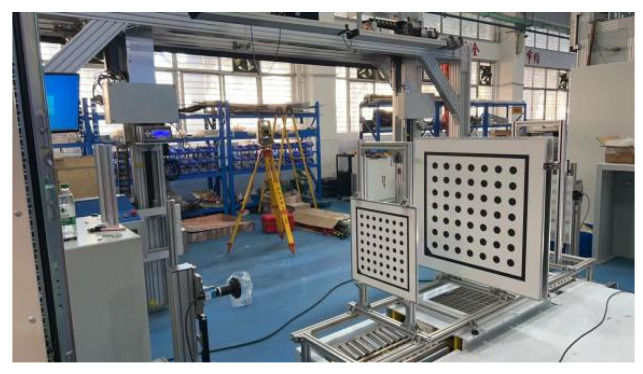
Calibrating Bracket Device.

**Figure 21:**
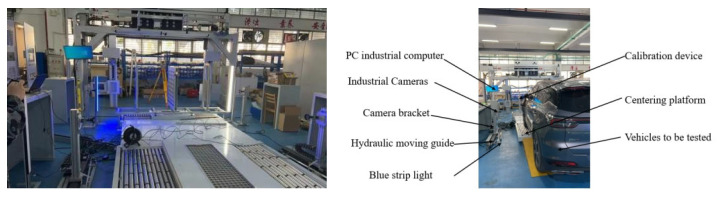
Structure Diagram of On-site Platform Device.

This study focuses on the wheel arch detection system designed in Section 3.1, enhanced with auxiliary equipment to improve experimental controllability and engineering applicability. Calibration fixtures were incorporated, and the platform setup, shown in Figure 20, follows three main principles: consistency with actual production line conditions, quantifiable parameter calibration, and alignment with workshop cost constraints. These principles ensure that the experimental results can be directly applied to the full-vehicle offline scenario. The overall platform structure is shown in Figure 21, with the camera positioned horizontally and the light source arranged at a 45° angle. The total hardware cost is ≤50,000 CNY, fully meeting the cost and space constraints of a complete vehicle offline workstation, while also enabling the simulation of various workshop lighting environments through adjustments to the light source power and camera exposure parameters.

### 4.2. Experimental Design and Data Collection

The experiment selected seven mainstream vehicle models as test subjects, including four SUVs with wheel arch ranging from 825 to 875 mm and three sedans with wheel arch ranging from 710 to 845 mm. For each model, 100 datasets were collected repeatedly. Environmental stability variables were controlled to compare detection performance across two different scenarios.

#### 4.2.1. Laboratory Simulation Scenario

This experiment is conducted in a standardized enclosed site to eliminate the influence of external factors on measurement results. The ground flatness is controlled within ≤0.5 mm/m, calibrated using a laser level to avoid vehicle attitude deviations caused by ground inclination. The lighting environment simulates natural light changes in a workshop during dawn and dusk using adjustable LED light sources, with an intensity fluctuation range set between 500 and 1500 lux. Throughout the experiment, no dust interference occurs, as industrial dust removal fans are used to maintain site cleanliness, ensuring stable image acquisition quality. The experimental procedure follows a standardized process:

1. Vehicle Positioning and Calibration: Seven vehicle models (A to G) are tested. After positioning through the guide wheel device on the side of the roller, the camera is calibrated using a camera calibration board to ensure that the camera’s imaging center is vertically aligned with the wheel hub center. The alignment deviation is controlled to ≤±0.2 mm.
2. Lighting Adjustment: The light source power is dynamically adjusted according to real-time light intensity. It is set to 15 W at 500 lux to compensate for low light and reduced to 8 W at 1500 lux to suppress reflection. The camera parameters, such as exposure time (500 μs) and gain (1 dB), are fixed to avoid image quality differences caused by parameter fluctuations.
3. Image Clarity Selection: For each vehicle model, three frames of front wheel images are collected at a single time. The clarity of the images is calculated using the Laplace operator [42], and clear images with a variance > 300 are selected, while blurry frames caused by vibration are eliminated.
4. Reference True Value Measurement: The wheel arch reference values are measured using the CMM (Coordinate Measuring Machine), with an accuracy of ±0.02 mm. The reference values for the models are as follows: Model A—765 mm, Model B—795 mm, Model C—845 mm, Model D—875 mm, Model E—825 mm, Model F—710 mm, Model G—805 mm.

100 sets of raw measurement data were randomly sampled for each model, and 10 sets of measurements were selected for representative analysis, as shown in Table 2. From the statistical results, it is evident that the measured values of each model fluctuate slightly around their corresponding reference values, with no obvious systematic deviation. For example, the average of 10 measurements for Model A is 764.833 mm, which shows a very small deviation from the reference value of 765 mm. Similarly, the average of 10 measurements for Model G is 804.881 mm, which is close to the reference value of 805 mm. The concentration and small fluctuation of the measured values suggest that the proposed detection method can maintain stable measurement output across different vehicle models, thus preliminarily validating the method’s basic effectiveness.

#### 4.2.2. On-Site Production Line Scenario

To further verify the applicability of the wheel arch detection method in an actual production line environment, the experiment was conducted in the off-line inspection area of a car manufacturing facility. The field environment was carefully set up to match the actual configuration of the production line workstation, with the following setup:

1. Lighting: The lighting system uses a combination of “workshop ceiling lights + LED fill lights,” with the intensity controlled at a constant 1000 ± 50 lux, ensuring no interference from natural light. This setup eliminates light fluctuations, which could otherwise affect image feature extraction.
2. Camera Setup: The camera is mounted horizontally at a preset angle relative to the production line roller track, ensuring that the wheel arch area appears in a standard projection form in the image.
3. Ground Setup: The ground is the standard roller track of the production line, calibrated with a laser level to ensure a flatness of ≤0.3 mm/m. This eliminates any vehicle attitude deviations caused by ground tilt, ensuring the consistency of the measurement reference.

The only environmental variable considered in this experiment was the removal of light fluctuation interference, leaving stable lighting conditions from the production line to isolate the impact of environmental changes on the results. This setup focused on verifying the stable output capability of the detection method in real-world industrial scenarios.

100 sets of raw measurement data were randomly sampled for each model, with 10 sets selected as representative samples, as shown in Table 3. The analysis results indicate that the average field measurement value for each model is closer to the reference value obtained using the CMM. For example, the average value of 10 measurements for Model F is 709.9809 mm, with a deviation of only 0.0191 mm. Similarly, the average value of 10 measurements for Model D is 875.0176 mm, with a deviation of only 0.0176 mm—both significantly smaller than the measurement deviations observed in the laboratory scenario.

Furthermore, the standard deviation of the measured values in the field scenario was ≤0.08 mm, which is notably lower than the standard deviation of ≤ 0.3 mm observed in the laboratory scenario. This indicates that the stable industrial environment significantly reduces measurement errors and improves data stability.

### 4.3. Error Quantification Analysis

The data are shown in Table 4, and the results indicate that the error in the laboratory scenario is primarily due to variations in image grayscale distribution caused by lighting fluctuations ranging from 500 to 1500 lux. Although these fluctuations are mitigated by the preprocessing steps described in Section 2.2, a residual fluctuation of ≤±1 mm remains. However, this level of fluctuation still meets the preliminary detection requirements for the vehicle end-of-line inspection phase.

**Figure 22:**
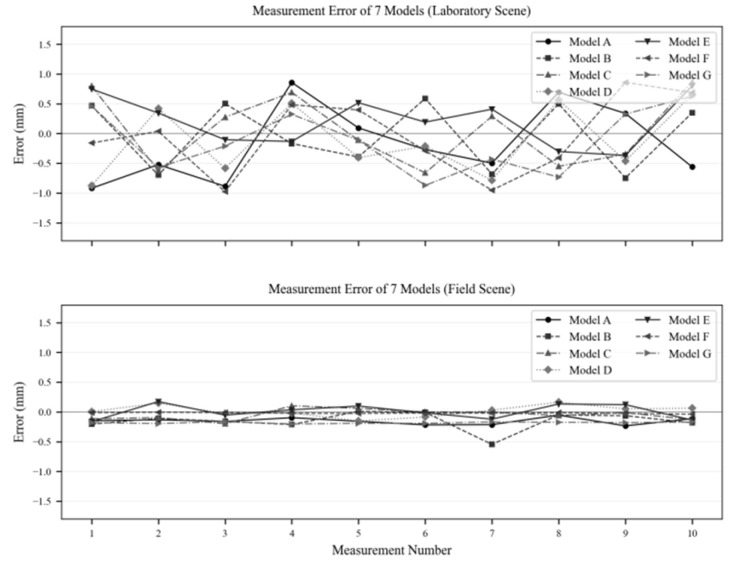
Error Distribution of Two Experimental Measurement Data.

The minimum Mean Absolute Error (MAE) is just 0.0004 mm (for Model F), and the maximum error is ≤±0.545 mm, which fully satisfies the ADAS calibration accuracy requirement of ±1.0 mm. The standard deviation (SD) is approximately 0.07 mm, demonstrating excellent data stability. The corresponding error distribution is shown in Figure 22. In the laboratory scenario, errors follow a wide normal distribution, whereas in the field scenario, errors are tightly concentrated within the ±0.2 mm range. This confirms that environmental stability plays a critical role in improving detection accuracy.

To assess the performance of the method in the production scenario of vehicles rolling off the production line and in complex environments, further verification of its adaptability and robustness was carried out:

Model Adaptation: For seven vehicle models, including four SUVs and three sedans, the error trends in both scenarios were highly consistent. The average Mean Absolute Error (MAE) for sedans (Models A, B, C, F) in the laboratory scenario was 0.321 mm, while the average MAE for SUVs (Models D, E, G) was 0.308 mm. The accuracy difference between the two categories of models was only 0.013 mm, showing no significant deviation. In the field scenario, the average MAE for sedans and SUVs further converged to 0.022 mm and 0.025 mm, respectively. Notably, there was no need to adjust algorithm parameters for individual models throughout the process, and no accuracy fluctuations occurred due to structural differences between models. This strongly confirms the method’s adaptability to mixed-line production.

Environmental Robustness: By comparing the core accuracy indicators between the two scenarios, the maximum deviation in the average measurement value of the same model was only 0.124 mm (e.g., Model B: laboratory 794.971 mm vs. field 794.847 mm), with no systematic deviation (fluctuation ≤ 0.005 mm). These results demonstrate that the method exhibits strong resistance to typical workshop interferences and can be stably adapted to real-world production environments.

### 4.4. Conclusions of Experimental Results

The core performance indicators of this wheel arch detection method fully meet industry standards. In real production scenarios, the mean absolute error (MAE) of wheel arch detection is ≤0.046 mm, with the maximum error ≤±0.545 mm, fully satisfying the precision requirements for vehicle offline ADAS calibration. The method demonstrates excellent vehicle model adaptability; for seven models, including four SUVs and three sedans, the detection error trends are highly consistent. The algorithm operates stably without requiring parameter adjustments, making it fully adaptable to mixed-line offline vehicle production.

The method also shows significant environmental robustness, maintaining stable detection performance even under typical workshop interferences, such as fluctuating lighting and dust. The average measurement deviation for the same model in both laboratory and field scenarios is ≤0.124 mm, with no systematic loss of accuracy.

Additionally, the method offers a strong cost advantage, with the total experimental platform hardware cost ≤50,000 CNY—much lower than the investment required for traditional laser profilometers—demonstrating economic feasibility for large-scale deployment.

In summary, this method comprehensively meets the core requirements of the vehicle offline process—“low cost, high precision, strong adaptability”—and serves as a reliable pre-detection solution for ADAS calibration.

## 5. Conclusions and Future Outlook

In response to the core requirements of “low cost, non-contact, high efficiency, and high precision” for wheel arch detection in the offline phase of vehicle production, this paper develops and validates an inspection system based on monocular machine vision. The total hardware cost is only 20,000 CNY, which is 90.3% lower than that of a laser profiler and 92.6% lower than a coordinate measuring instrument. Additionally, the 5-year lifecycle cost is 30.4% of the manual tape measure approach. Testing across seven vehicle models (3 cars and 4 SUVs) resulted in an MAE ≤ 0.25 mm, with a maximum error ≤ 0.48 mm, meeting the ADAS calibration requirement of ±0.5 mm. The system detects all four wheel arches of a single vehicle in ≤3.2 s, meeting the industry standard cycle time of 60 jobs per hour (JPH), and shows an MAE fluctuation of ≤0.08 mm in environments with 500–1500 lux lighting and ≤10 mg/m^3^ dust. The non-contact design also prevents body paint damage. Relying on open-source technology, the system can be deployed within 3 days and supports expansion to over 20 vehicle models.

However, the system has some limitations: it currently supports only stationary vehicle detection (with error increasing to ±0.8 mm when the vehicle is dynamically conveyed at 0.5–1 m/s). Additionally, feature extraction stability is reduced under extreme conditions (strong light > 2000 lux, high humidity RH > 85%). Future improvements will include the Lucas-Kanade optical flow method for dynamic detection at speeds up to 1 m/s (error ≤ 0.5 mm), integration of a low-cost TOF laser module to extend environmental adaptability (50–3000 lux illumination, 30–90% RH), and optimization of the YOLOv5s model via transfer learning to reduce the adaptation time for new models from 2 h to 10 min, further enhancing the system’s adaptability in mixed-line production.

## Figures and Tables

**Table 1:** Hub Measurement Comparison Parameters.

	Row	Column	Radius	Measured Value/mm	Reference Value/mm
Threshold method	1151.28	620.446	506.697	498.705	498
MSER method	1149.01	621.921	505.586	497.609	498
Canny method	1149.39	622.056	506.498	498.508	498

**Table 2:** Experimental Data of Front Wheel Measurements for 7 Vehicle Types at the Test Site.

	A Measurement Value/mm	B Measurement Value/mm	C Measurement Value/mm	D Measurement Value/mm	E Measurement Value/mm	F Measurement Value/mm	G Measurement Value/mm
1	764.08	795.466	845.798	874.125	825.747	709.841	805.462
2	764.477	794.301	844.373	875.42	825.344	710.036	804.428
3	764.113	795.502	845.274	874.416	824.896	709.021	804.789
4	765.858	794.832	845.692	875.508	824.867	710.483	805.323
5	765.094	794.606	844.887	874.594	825.518	710.397	804.887
6	764.731	795.589	844.338	874.789	825.192	709.71	804.128
7	764.502	794.318	845.289	874.213	825.409	709.048	804.568
8	765.694	795.499	844.449	875.567	824.697	709.589	804.267
9	765.338	794.253	844.651	874.538	824.632	710.86	805.326
10	764.439	795.348	845.872	875.656	825.801	710.688	805.629
Average value/mm	764.833	794.971	845.062	874.883	825.210	709.967	804.881

**Table 3:** Field Site Experimental Data of Front Wheel Measurements for 7 Types of Vehicles.

	A Measurement Value/mm	B Measurement Value/mm	C Measurement Value/mm	D Measurement Value/mm	E Measurement Value/mm	F Measurement Value/mm	G Measurement Value/mm
1	764.851	794.794	844.884	875.005	824.836	709.988	804.819
2	764.868	794.891	844.905	875.148	825.174	709.995	804.805
3	764.840	794.841	844.802	874.973	824.946	709.989	804.837
4	764.903	794.788	845.103	874.982	825.036	709.977	804.798
5	764.842	795.010	845.061	874.849	825.099	709.975	804.808
6	764.783	794.992	844.999	874.913	824.985	709.981	804.809
7	764.787	794.455	844.997	875.029	824.878	709.980	804.832
8	764.946	794.949	844.940	875.164	825.133	709.987	804.827
9	764.766	794.936	844.988	875.051	825.122	709.976	804.820
10	764.886	794.817	844.869	875.062	824.863	709.961	804.829
Average value/mm	764.8472	794.8473	844.9548	875.0176	825.0072	709.9809	804.8184

**Table 4:** Error Analysis Parameters.

Scene	Laboratory Scene			On-Site Scenario		
Indicator	MAE (mm)	Max Error (mm)	SD (mm)	MAE (mm)	Max Error (mm)	SD (mm)
A	0.388	±0.92	0.612	0.026	±0.234	0.071
B	0.288	±0.747	0.589	0.046	±0.548	0.082
C	0.33	±0.872	0.595	0.01	±0.198	0.069
D	0.334	±0.875	0.603	0.009	±0.164	0.067
E	0.205	±0.801	0.578	0.014	±0.174	0.073
F	0.375	±0.979	0.621	0.0004	±0.039	0.065
G	0.269	±0.872	0.591	0.033	±0.202	0.07

## Data Availability

The original contributions presented in this study are included in the article. Further inquiries can be directed to the corresponding author.
